# Potential roles of acyl homoserine lactones (AHLs) in nitrifying bacteria survival under certain adverse circumstances

**DOI:** 10.1038/s41598-022-23123-x

**Published:** 2023-02-06

**Authors:** Xiangguo Zeng, Huizhi Hu

**Affiliations:** 1 Wuhan Planning and Design Co., LTD, Wuhan, 430010 China; 2 Faculty of Resources and Environmental Science, Hubei University, Wuhan, 430062 China; 3 Hubei Key Laboratory of Regional Development and Environmental Response, Wuhan, 430062 China

**Keywords:** Biotechnology, Environmental sciences

## Abstract

Potential roles of quorum sensing (QS) in nitrifying bacteria activity and ecology, particularly under adverse circumstances have been rarely reported. Herein, eight lab-scale nitrification sequencing batch reactors, with or without adding acyl homoserine lactones (AHLs) were operated under adverse circumstances respectively. The results indicated that the introduction of AHLs significantly enhanced the nitrogen removal efficiency in the presence of nitrification inhibitors (dicyandiamide, DCD), accelerated the low temperature (10 °C) group into stable stage, and improved the utilization efficiency of AHLs in these two groups. Community analysis and qPCR further confirmed that AHLs significantly increased the abundance of nitrifying bacteria in low temperature group and DCD group, especially AOB. For normal condition (28 °C, pH = 8) or low pH level (5.5), however, the AHLs had no significant effect. Canonical correspondence analysis showed that nitrifying bacteria positively responded to AHLs, indicating that adding AHLs was an effective strategy to regulate nitrification process. However, under acid conditions, the effect of this regulatory mechanism was not significant, indicating that the influence of pH on the system was greater than that of AHLs. This study demonstrated that exogenous AHLs could enhance the competitiveness of nitrifying bacteria to utilize more resource and occupy space under some adverse environmental conditions.

## Introduction

Nitrifying bacteria are kinds of relatively weak autotrophic aerobic bacteria, with longer generation cycle and higher requirements on the survival environment. Some adverse factors can influence the activity of the nitrifying bacteria, such as low temperature (< 15 °C), pH, short hydraulic retention time (HRT), free ammonia (FA) and free nitrous acid (FNA)^1,2^. In addition, there may be some trace nitrification inhibitors (NI) in the influent of sewage treatment plant, such as dicyandiamide (DCD, a common ammonia oxidation inhibitor that is frequently reported in real municipal wastewater treatment plants)^3^, which can significantly inhibit the activity of nitrifying bacteria. Some change of environmental factors also have a significant impact on the nitrification process, and it will take a long time for the system to realize stable operation again. As a consequence, it is of great practical significance to explore the rapid recovery of adverse effects on nitrification system.

Quorum sensing (QS) regulate the ecological relationship of flora and physiological behavior by releasing and sensing the concentration of signal molecule to induce the expression of related genes in bacteria, and achieve the physiological functions and regulatory mechanisms that single bacteria can't realize, such as biofilm formation and the secondary metabolites production, etc.^4–6^. Bacteria rely on the physiological activities above to survive under environmental stresses^7^. *N*-acyl homoserine lactones (AHLs), one of QS signaling substances, has been well-characterized in Gram-negative bacteria^8,9^. At present, many nitrifying bacteria in both pure and mixed cultures have QS effect, and their correlation with QS has been confirmed by gene sequencing technology^10,11^. Pure cultures of many ammonia-oxidizing bacteria (AOB) supernatant solutions could produce AHLs, such as *Nitrosomonas europaea* and *Nitrosospira multiformis*^11,12^*.* AHLs were also detected in membrane bioreactor (MBR), nitrifying biofilms and autotrophic nitrifying biofilms^13–15^. Although some AOB do not produce AHL (with AHL receptor gene, without AHL synthase gene), they can use exogenous AHL^12^. *Nitrosospira multiformis*, another AOB model organism, has recently been found to have LuxI/R type QS signaling synthase and regulator, which can produce C4-HSL and 3-o-C14-HSL signaling molecules^16^. Yu et al. found that enhance quorum quenching to inhibit QS in MBR would reduce the nitrification effect, proving the importance of QS to nitrification indirectly^17^. These studies showed a strong relationship between nitrifying bacteria and QS. Thus, it is reasonable to suspect that QS, which is great important for bacteria to survive under environmental stresses, may improve nitrifying bacteria activity and nitrogen removal under stressful conditions.

To evaluate the potential regulatory roles of AHLs on bacteria behavior and characteristics of nitrifying bacteria under certain adverse conditions, including low temperature, low pH level and presence of DCD, eight batch tests with or without AHLs adding were conducted. The types of AHL signal molecules added were C6-HSL and C8-HSL, respectively, which were dominant in the system according to the detection results. The nitrogen removal ability and QS activity dosed with AHLs were examined, and the number of AOB& nitrite-oxidizing bacteria (NOB) as well as bacterial community and diversity were analyzed, thus the possible mechanism of exogenous signals on nitrifying microorganisms was proposed. The aim of this study is to investigate the effect of AHLs on nitrogen removal, and finally proposing a strategy to enhance nitrifying bacteria activity under some stressful conditions.

## Materials and methods

### Chemical reagent

Signaling molecules used in this study (*N*-Hexanoyl-l-homoserine lactone (C6-HSL) and *N*-octanoyl-l-homoserine lactone (C8-HSL)) were purchased from Sigma-Aldrich (China). AHLs were dissolved in methanol with a final concentration of 1 g/L as the stock solutions, and formic acid was added at 0.1% concentration (v/v). DCD was purchased from Sigma-Aldrich.

### Inoculum preparation

One fully automatic sequencing batch reactor (SBR) with an effective volume of 6 L was used for nitrifying bacteria inoculum preparation. The reactor cycle time was 8 h comprising of the following phases: 30 min feeding, 4 h aeration, 2 h anoxic, 1 h settling and 30 min decanting. Seed sludge was obtained from the return sludge of settling tank in Wuhan wastewater treatment plant. Details of synthetic feed stock solution are shown in Supplementary Table S1. The concentrations of NH_4_^+^–N was initially 25 mg/L, increased to 50 mg/L after two weeks culture and remained for two weeks, and then raise to the final concentration of 100 mg/L. pH level was maintained at 8.0 by Na_2_CO_3_. The dissolved oxygen (DO) concentration was varied around 2.0–4.0 mg/L (aeration) using an air flow meter. Temperature was in the range of 28 °C with a thermostatic heater (GDH-4006, SCIENTZ). The nitrifying bacteria were harvested when approximately 65% of the ammonium was oxidized, and then detected AHL in the supernatant.

### Batch test

Eight 1000-mL automatic SBRs filling with 300 mL synthetic feed stock solution and 300 mL nitrifying bacteria inoculum, divided into four groups averagely, normal group (N-BR and N-ER), acid group (A-BR and A-ER), DCD group (D-BR and D-ER) and low temperature group (L-BR and L-ER). The flask filled with 1 μM AHLs mixtures (C6-HSL and C8-HSL iso-volume mixing) was experiment reactor (ER), and the flask without AHLs was blank reactor (BR). The reactors operate in the same manner as in “Inoculum preparation”. The details of four groups are shown in Supplementary Table S2. The influent NH_4_^+^–N concentration is 100 mg/L. The added types of AHLs were confirmed according to dominant types in reactors, and the added concentration of AHLs was determined according to relevant literatures^10,17^. AHLs mixture was added to flask once per day during the feeding phase. All flasks were operated as mentioned in 2.2 for 20 days.

### Analytical methods

Ammonia nitrogen (NH_4_^+^–N), nitrite nitrogen (NO_2_^–^N) and nitrate nitrogen (NO_3_^–^N) were measured according to standard methods per two days (APHA, 2005), and the removal efficiency of each index was calculated according to Eq. (1). Mixed liquor suspended solids (MLSS) was defined as drying at 105 °C for 12 h once per two days. DO and pH were measured by a multi-parameter water quality analyzer (HQ30D, HACH, USA). The free ammonia (FA) and free nitrous acid (FNA) concentrations were calculated according to Eq. (2) and (3). SPSS software (version 19.0) was applied to carry out T test to test the differences among various samples, and it was considered the differences were statistically significant when *p* < 0.05.

1 $$ {\text{Removal}}\,{\text{efficiency (\% ) }} = \, \frac{{{\text{influent}}\,{\text{concentration - effluent}}\,{\text{concentration}}}}{{{\text{influent}}\,{\text{concentration}}}} \, \times {100,} $$

2 $$ {\text{FA}}\,{\text{as}}\,{\text{NH}}_{{3}} \,{\text{(mg}}\,{\text{L}}^{ - 1} {) } = \, \frac{{{17}}}{{{14}}} \times \frac{{[{\text{NH}}_{4}^{ + } - {\text{N}}]({\text{mg}}\,{\text{L }}^{ - 1} ) \times 10^{{{\text{pH}}}} }}{{e^{{[6344/(273 + T(^\circ {\text{C}}))]}} + 10^{{{\text{pH}}}} }}, $$

3 $$ {\text{FNA}}\,{\text{as}}\,{\text{HNO}}_{{2}} \,{\text{(mg}}\,{\text{L }}^{ - 1} {)} = \frac{{{46}}}{{{14}}} \times \frac{{[{\text{NO}}_{{2}}^{ - } - {\text{N}}]({\text{mg}}\;{\text{L}}^{ - 1} )}}{{e^{{[ - 2300/(273 + T(^\circ {\text{C}}))]}} \times 10^{{{\text{pH}}}} }}. $$

### AHL detection and degradation test

AHL in nitrifying bacteria inoculum was extracted from supernatants and concentrated by solid-phase extraction (SPE) using C18 columns (Agilent, America) according to Li et al.^18^. AHL concentration was analyzed by ultra-performance liquid chromatography (UPLC) equipped with an electrospray ionization source (ESI). The detailed methods for standard AHLs were according to our previous study^19^.

To evaluate the degradation of AHL in the system, 500 mL activated sludge were removed from N-BR and A-BR and inactivated in the beakers, separately. Then 1 μM of C6-HSL and C8-HSL were added to the beakers. The mixture were incubated in a shaker at 28 °C, 120 rpm for 24 h. Periodically remove the mixture from the beakers to measure the concentration of AHL.

### High-throughput sequencing analysis for community composition

DNA extraction was conducted with a DNeasy PowerSoil Pro Kit (QIAGEN, Germany). Nucleic acid concentrations were determined by spectrophotometer (NanoDrop). Sequencing of 16S rDNA was carried out on Illumina HiSeq 2500 system by Sangon Biotech (Shanghai, China). The primer sequences were 338F (5′-ACTCCTACGGGAGGCAGCAG-3′) and 806R (5′-GGACTACHVGGGTWTCTAAT-3′) with a final concentration of 5 μmol/L.

### Real-time PCR analysis

Total DNA extraction was carried out as mentioned in “High-throughput sequencing analysis for community composition”. The primers used to amplify 16s rRNA gene, amoA gene and nxr gene are shown in Supplementary Table S3. Establishment of amplification procedure and formulation of reaction system were conducted according to PerfectStart Green qPCR SuperMix (TransGen Biotech, China) instructions. The final volume of each PCR reaction was 20 μL, which contained as follows: SYBR Green PCR master mix (Applied Biosystems, USA) 12.5 μL, the template DNA (the extracted DNA was diluted 100 times) 3 μL, each of forward and reverse primer 1 μL, as well as ddH_2_O 2.5 μL. Then, the PCR amplification was performed by a sequence detection system (ABI Prism 7500). qPCR used a two-step method: 30 s of denaturation at 94 °C, 40 cycles of 5 s at 94 °C for and 60 °C for 30 s. Melt curve analysis was performed at the end of all qPCR runs to determine the specificity of amplified products.

## Results

### Impact of AHLs on nitrification under adverse circumstances

**Figure 1:**
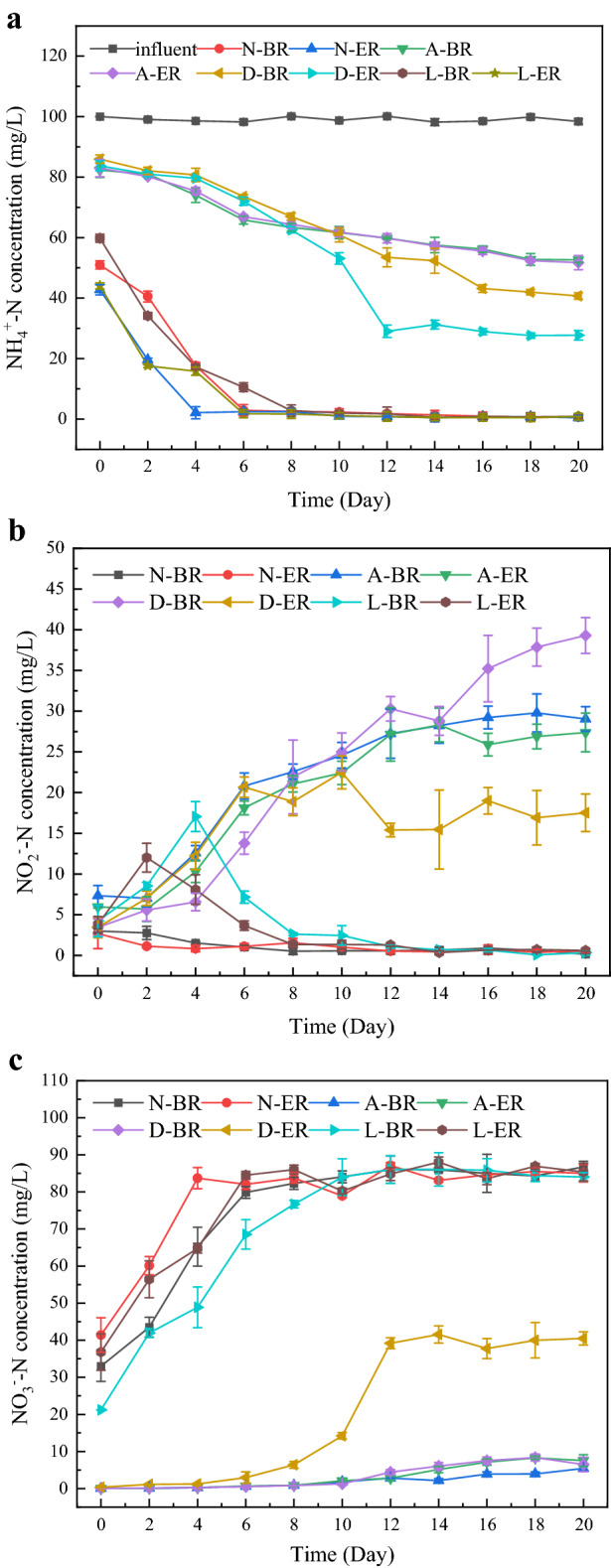
The performance of the activated sludge during the operation period of four groups. *N* normal group, *A* acid group, *D* DCD group, *L* low temperature group, *BR* blank reactor, *ER* experiment reactor. Error bars are defined as standard error of the mean (n = 3, biological replicates).

**Figure 2:**
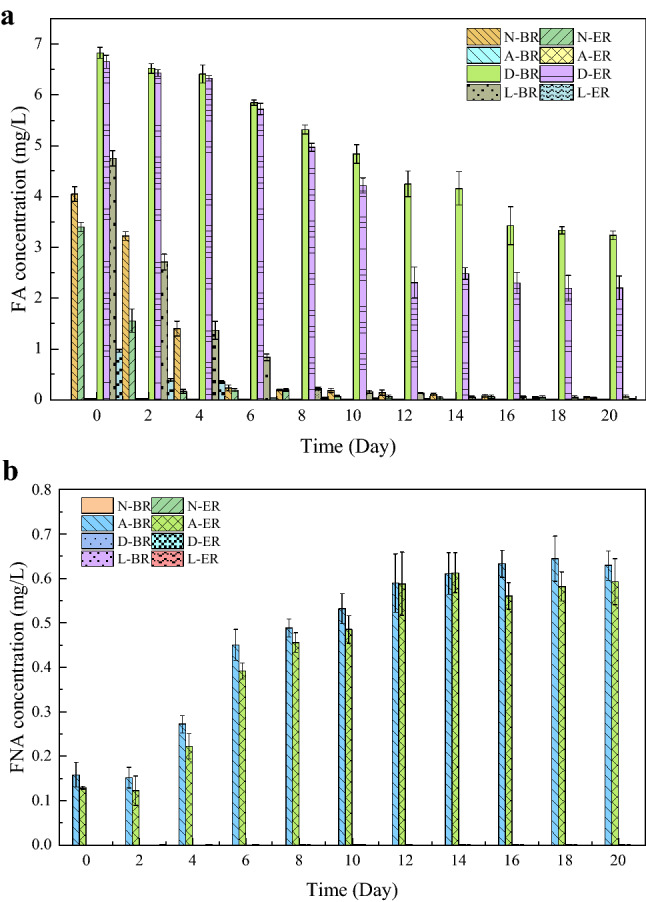
FA and FNA levels of four groups of activated sludge during operation. *N* normal group, *A* acid group, *D* DCD group, *L* low temperature group, *BR* blank reactor, *ER* experiment reactor. Error bars are defined as standard error of the mean (n = 3, biological replicates).

The initial NH_4_^+^–N concentration was around 99.08 ± 0.52 mg/L. The ammonia removal trend over time were quite similar in all tests except N-BR and N-ER, in which the removal efficiencies were slightly faster than other groups in the first 2 days (Fig. 1a). This was followed by a striking increase of ammonia removal efficiency in all tests. The NH_4_^+^–N removal efficiency in N-ER and L-ER was approximately 99% at the end of experiment, which was no significant increased compared with N-BR and L-BR (*p* = 0.055), except that the time of entering the stable stage was earlier than that of BR, indicating the reliance of AOB on QS was not strong in such environment. Culturing under unfavorable environment for 20 days, NH_4_^+^–N removal efficiency in DCD group and acid group was significantly lower than that in normal group. The NH_4_^+^–N removal efficiency between reactors of acid did not showed significant differences (*p* = 0.649), which were around 47% for BR and 48% for ER at the end of experiment, respectively. The lower ammonia conversion efficiency may be attributed to the effect of FNA. FNA concentration in acid group was in the range of 0.15–0.64, higher than the inhibition threshold of 0.02 mg/L^24^, whereas FNA in other groups was less than the inhibition threshold (Fig. 2a). However, for the test in DCD group, a change of NH_4_^+^–N removal was presented in reactors with exogenous AHLs addition (*p* < 0.05), with final NH_4_^+^–N removal efficiency of 59% (BR) and 72% (ER), respectively.

Effect of AHLs addition on NO_2_^–^N and NO_3_^–^N were also examined in all batch tests (Fig. 1b,c). Obviously, formation of NO_2_^–^N and NO_3_^–^N happened with a decrease in NH_4_^+^–N. Due to the lower FA concentration (< 6 mg/L) and FNA concentration (< 0.02 mg/L) in the normal group (Fig. 2a,b), the NO_2_^–^N level decreased accompanied by NO_3_^–^N level increased sharply. At the end of experiment, the concentration of NO_3_^–^N was around 86 mg/L. These results were consistent with NH_4_^+^–N removal efficiency. Some researchers have been proven that NOB was less tolerant to FA and FNA^24^. It would be inhibited when the FA level was above 6 mg/L or FNA concentration was more than 0.02 mg/L. Therefore, the NOB in normal group was not inhibited by FA and FNA, and almost all nitrite converted to nitrate. The changes of NO_2_^–^N and NO_3_^–^N concentration in acid-group two reactors were similar and stabilized at 27–29 mg/L and 5–7 mg/L approximately, indicating that the activity of NOB was inhibited. The NO_2_^–^N and NO_3_^–^N showed significant differences in D-BR and D-ER. Concentrations of the effluent NO_2_^–^N and NO_3_^–^N were 39.3 ± 2.21 and 6.51 ± 1.95 mg/L in D-BR, and 17.52 ± 2.31 and 40.49 ± 1.79 mg/L in D-ER. These results indicated that the addition of AHL effectively alleviated the inhibitory of DCD on NOB and promoted the transformation of NO_2_^–^N. In low temperature group, NO_2_^–^N accumulation occurred in both reactors at the beginning of the experiment, and then gradually transformed into NO_3_^–^N, while L-ER relieved NO_2_^–^N accumulation earlier than L-BR. During this process, FNA and FA concentrations reach the level below inhibition threshold of NOB, leading to the lower NO_2_^–^N concentration in L-ER. In addition, the significant different of NO_2_^–^N and NO_3_^–^N levels between DCD group and low temperature group might attribute to the AOB activities as mentioned above.

### Impact of AHLs addition on bacteria using AHL

**Figure 3:**
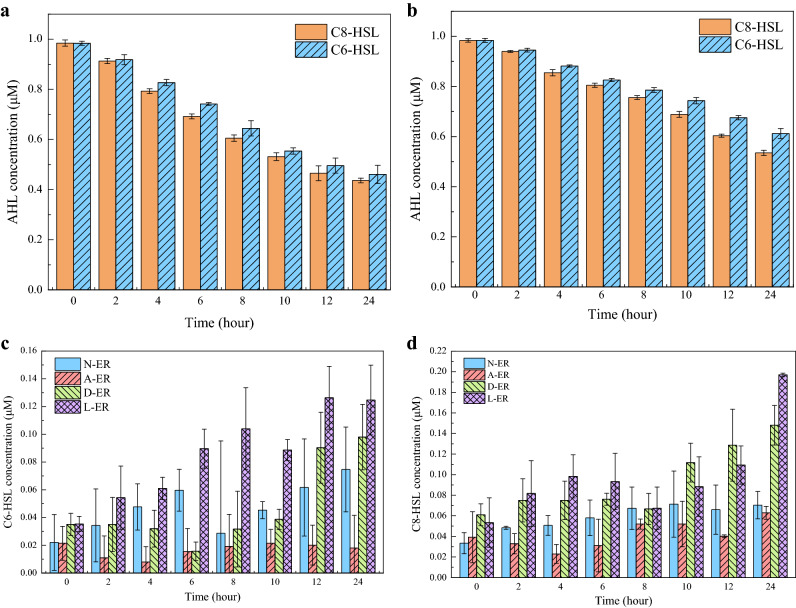
Detection of the degradation and usage of AHL in sludge supernatant sample in four groups reactors by UPLC-MS/MS. (**a**) The degradation of AHL in 24 h when pH = 8.0; (**b**) the degradation of AHL in 24 h when pH = 5.5; (**c**) the usage of C6-HSL in four AHL addition reactors; d: the usage of C8-HSL in four AHL addition reactors. *N* normal group, *A* acid group, *D* DCD group, *L* low temperature group, *ER* experiment reactor. Error bars are defined as standard error of the mean (n = 3, biological replicates).

Five types of AHLs were detected in the initial wastewater, namely C4-HSL, C6-HSL, C7-HSL, C8-HSL and 3-oxo-C12-HSL. Among them, C6-HSL and C8-HSL were dominant in the system, and they have been confirmed to be widely present in nitrifying bacteria system and other water treatment facilities, so this experiment mainly studied these two AHLs^12,20^. Concentrations of C6-HSL and C8-HSL were stable during the test, range between 13.34 and 34.85 nM in all batch conditions. In order to determine the utilization of AHL by bacteria under different conditions, the degradation of AHL in 24 h were detected firstly as a blank control. As can be seen from the Fig. 3a,b, the degradation efficiency of C6-HSL was significantly lower than that of C8-HSL under the same conditions, and the AHL level under acidic conditions was significantly higher. At the end of experiment, C6-HSL degraded 53% (pH 8.0) and 38% (pH 5.5), and C8-HSL was 56% (pH 8.0) and 46% (pH 5.5). Since the activated sludge had been inactivated, the degradation of signal molecules may due to the adsorption of activated sludge. This is consistent with the investigations of Tan et al.^21^. In addition, difference degradation efficiency between pH 8.0 and pH 5.5 might be explained by the easier degradation of AHL under alkaline conditions^22^.

After two kinds of AHL were added to different systems for 20 days, the degradation efficiency of AHL in the system changed (Fig. 3c,d). The original concentration was ignored because the added concentration was much higher than the generated by system (13.34–34.85 nM). In N-ER, the degradation efficiency of C6-HSL and C8-HSL changed to 60% and 62%, with an increase of 11.7% and 9.7%. The increased degradation efficiency suggested that part of added AHL was utilized by bacteria. In the acid environment (pH 5.5), the degradation efficiency of C8-HSL increased slightly from 46 to 50%, while the degradation efficiency of C6-HSL remained at about 38%. As can be seen from the experimental results, bacterial growth was inhibited under acidic conditions, resulting in a low AHL utilization efficiency, indicating that QS activity of this system was low. Noticeable changes in DCD group and low temperature group were also detected. The degradation efficiencies of two AHLs increased distinctly, with 62% of C6-HSL and 69% of C8-HSL in D-ER, and 65% of C6-HSL and 74% of C8-HSL in L-ER respectively, indicating more AHLs were utilized by bacteria. The above results suggested that AHL could be correlated with activities of nitrifying bacteria, which was consistent with the result of nitrogen removal process.

### Impact of AHLs addition on the relative abundance of nitrifying bacteria

**Figure 4:**
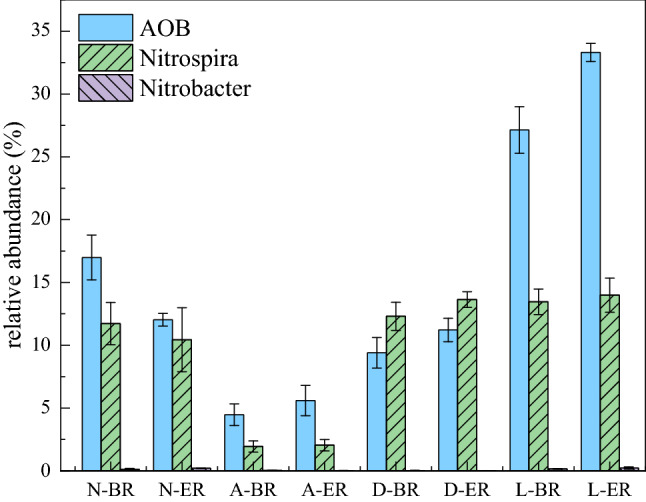
The abundance ratio of AOB and NOB to total bacteria in four groups. *N* normal group, *A* acid group, *D* DCD group, *L* low temperature group, *BR* blank reactor, *ER* experiment reactor.

Considering that the addition of AHLs significantly altered the nitrogen removal efficiency of batch reactor, it was necessary to analyze the effect of exogenous AHLs on the activity of nitrifying bacteria. As shown in Fig. 4, the abundance of AOB and *Nitrospira* were consistently dominant in all groups. The percentage of AOB and *Nitrospira* in the total bacteria showed a slight decline trend in normal group after adding AHLs, AOB from 16.98% (BR) to 12.03% (ER), *Nitrospira* from 11.73% (BR) to 10.44% (ER), respectively, indicating that AHLs did not increase the abundance of nitrifying bacteria. Similarly, the addition of AHLs did not obviously reverse both of AOB and *Nitrospira* numbers decline at pH 5.5, since the abundance of AOB and NOB were 4.46% and 1.94% in BR, and 5.09% and 2.04% in ER, respectively. However, after adding 1 μM AHL, AOB and NOB biomass in DCD and low temperature group increased significantly, especially AOB with the proportion increased from 9.4% (D-BR) and 27.14% (L-BR) to 11.22% (D-ER) and 33.31% (L-ER), respectively. At the same time, there was no significant incrased in total bacteria biomass in DCD and low temperature group (data not shown). This clear increase in AOB was considered to be an important effect of AHLs on the system. It is worth noting that the abundance of nitrifying bacteria at low temperatures was generally higher than that of the normal group, especially AOB, which further reflected the different regulation strategies of AHLs at room temperature and low temperature. Considering the performance of the reactor, it was inferred that AHLs tended to increase the abundance of nitrifying bacteria in response to environmental pressures at low temperatures.

### Bacterial diversity and community structure responding to different treatment processes

From Table 1, the high Good's coverage of samples (more than 98%) indicated the result represents the microbial components of sludge samples. Higher Chao1 and ACE values were observed in experimental reactor of four groups, which means greater richness. Shannon and Simpson of the samples changed during the cultivation of nitrifying bacteria in different environments. Species diversity decreased significantly in the acid group, but increased significantly in the low temperature and DCD groups. Notably, the diversity index in AHL addition reactor were higher than that of in control reactor among 3 groups (expect acid group).

**Table 1:** Sequencing information and diversity index under 97% similarity.

	N-BR^a^	N-ER	A-BR	A-ER	D-BR	D-ER	L-BR	L-ER
Coverage	0.9899	0.9901	0.9846	0.9896	0.9932	0.991	0.9928	0.9949
Chao1	1847	1895	1277	1169	1632	1652	1469	1578
ACE	1874	1933	1426	1362	1653	1724	1523	1605
Shannon	4.243	4.734	3.920	3.849	5.031	5.244	5.372	5.566
Simpson	0.03797	0.03595	0.04084	0.04181	0.02089	0.02011	0.01687	0.01590

^a^*N* normal group, *A* acid group, *D* DCD group, *L* low temperature group, *BR* blank reactor, *ER* experiment reactor.

**Figure 5:**
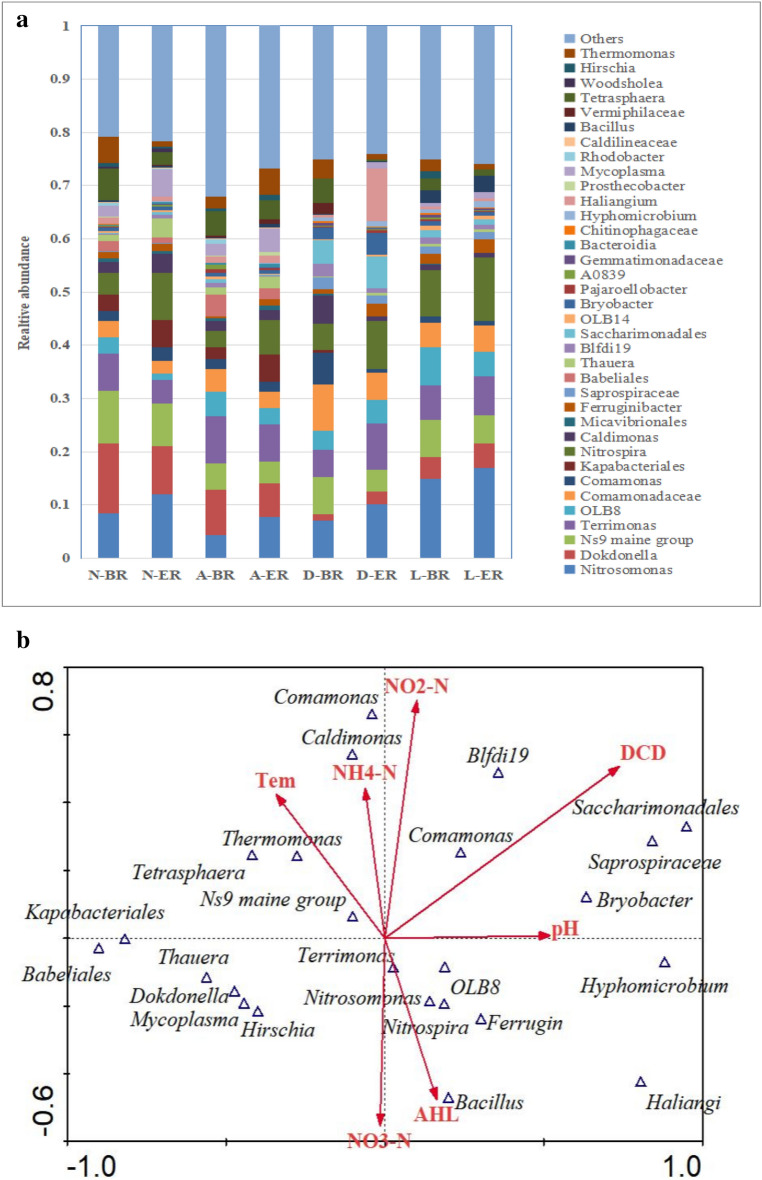
Bacterial compositions and canonical correspondence analysis between the environmental factors and community among 8 reactors. (**a**) distributions of bacterial community in the sludge in different reactors; (**b**) canonical correspondence analysis between the environmental factors and bacterial community. *N* normal group, *A* acid group, *D* DCD group, *L* low temperature group, *BR* blank reactor, *ER* experiment reactor.

Comparison of high-throughput sequencing profiles from 8 reactors showed significant differences in bacterial compositions (Fig. 5a). A total of 36 bacteria genera across all samples with RDP classifiers were identified from our filtered sequence. *Nitrosomonas*, *Dokdonella*, *Ns9 maine group*, *Terrimonas*, *Tetrasphaera*, and *Thermomonas* were the dominant bacteria accounting for approximately 5% to 13% (relative abundance) of the bacteria community. After 20 days of cultivation with AHLs, the relative abundance of *Nitrosomonas* and *Nitrospira* both increased by more than 20%, in which *Nitrospira* obtained the highest relative abundance of 53.9%, 53.8%, 43.8% and 28.3%, respectively. *Nitrosomonas* is commonly found in wastewater treatment plants with low ammonium levels. Higher relative abundance for this genus was usually achieved when NH_4_^+^–N was lower. *Nitrospira* is the main nitrite oxidizing bacteria existing in sewage treatment plants and laboratory reactors. The changes of these two bacteria abundance were consistent with changes in nitrogen degradation efficiencies described in “Impact of AHLs on nitrification under adverse circumstances”. It was worth noting that the abundance of these two bacteria was significantly higher at low temperature than at normal temperature, consistent with the results in “Impact of AHLs addition on the relative abundance of nitrifying bacteria”. The abundance of *Ns9 maine group* decreased significantly in all experimental groups, suggesting that this genus may be inhibited by AHLs. The significant variation of these genus might be a result of AHLs feeding, indicating that exogenous AHLs could be a critical factor in altering bacteria community.

The variation of bacterial community, which influence by water quality and environmental factors, such as pH, temperature and nitrification inhibitors, was explained by Canonical correspondence analysis (CCA). The biplot diagram highlighted relationships between environmental factors and composition of bacterial with a species weight > 5% during four cultivation processes in Fig. 5b. DCD and NO_2_^–^N concentration had the greatest effect on the distribution of bacterial community, while NH_4_^+^–N concentration had the least effect, which also explained the high degradation efficiency of NH_4_^+^–N under the four operating conditions, while NO_2_^–^N accumulated in some groups. Most of the dominant bacteria in the system (*Dokdonella*, *Ns9 maine group*, *Terrimonas*, *Tetrasphaera*, and *Thermomonas*) were significantly negatively correlated with pH, resulting in poor system treatment when operating at low pH. It was worth noting that there was a significant positive correlation between nitrifying bacteria and AHL, indicating that the addition of AHL can significantly promote the nitrification process. Thus, the results confirmed that the nitrifying bacteria community was both gradually and highly affected during the AHL addition period.

## Discussion

In biological wastewater treatment facilities, QS regulate bacteria density and organize their collective behavior, which also has important effects on activated sludge, microbial community and reactor performance. Many studies have confirmed that many physiological functions of nitrifying bacteria in WWT are related with AHL-based QS^10–15^. The detection of signal molecules further confirmed this conclusion. Several types of AHLs generated by AOB had been demonstrated in supernatant in previous studies^23^. However, the available reports for the actions of AHLs-mediated QS in nitrifying bacteria are very limited, especially the nitrifying bacteria in unfavorable growth conditions. Whether QS can promote the recovery of nitrifying bacteria activity needs further study. Thus, we attempted to estimate the potential impact of AHLs on the regulation of bacteria behavior and characteristics of nitrifying bacteria under adverse condition. Our data demonstrated a significant promotion of AHLs on nitrifying bacteria under unsuitable conditions. The positive roles of AHLs mediated QS in the presence of DCD and at low temperatures has been found, under which the proportion of AOB and NOB in all bacteria increases significantly, thus improving the efficiency of the system under adverse conditions. However, the effect was not significant under normal culture condition and acid condition.

Environmental factors would have a profound impact on the effectiveness of AHLs, thus affecting the level of AHL-based QS in surrounding environment. When pH is not conducive to AOB and NOB growth, the effect of pH on AOB was stronger than AHLs on AOB, so the promotion effect of exogenous AHL is not significant. The NH_4_^+^–N removal efficiency and the formation efficiency of NO_2_^–^N and NO_3_^–^N between low pH systems almost kept at a lower level. Meanwhile, cultivation of sludge in pH 5.5 demonstrated that adding AHL does not obviously reverse both of total bacteria and AOB&NOB numbers decline. The different NH_4_^+^–N and NO_2_^–^N conversion efficiency between the low pH system and other systems may be attributed to the effect of FNA. Previous studies indicated that the biosynthesis of AOB was inhibited when FNA level reached 0.1 mg/L, and NOB was more sensitive to FNA which became inhibited when FNA level reached 0.02 mg/L^24^. Combined with the experimental results of 3.1 and 3.2, it can be seen that the high concentration of FNA under acid condition inhibited the growth of bacteria. FNA concentration was in the range of 0.13 to 0.64 in the acid system, higher than the inhibition threshold as mentioned above, whereas FNA in other groups were less than 0.02 mg/L. When the surrounding environment was suitable for the growth of bacteria, the activity of bacteria and the concentration of signal molecules secreted by the bacteria were both in the appropriate range. Therefore, the promotion of exogenous AHL on the bacteria was not significant, and there was no significant difference between N-BR and N-ER (*p* < 0.05). When the system was at low temperature or in the presence of inhibitors, the activity of nitrifying bacteria was inhibited, and the utilization efficiency of AHL reduced accordingly. After adding AHLs, the activity of nitrifying bacteria increased and the use of the surrounding signal molecules was more sufficient, so that the proportion of AOB and NOB in the total bacteria population increased, indicating that AOB and NOB gradually dominates in the competition with the symbiotic bacteria. This was as also shown by Li et al. that the ammonia removal had similar effect dosing with AHLs^10^. The QS system of microorganisms usually plays an important role in the rational utilization of resources and space^25,26^. QS system could regulate the growth of nitrifying bacteria, and then occupied more space and resources, making AOB and NOB become the dominant strain under some adverse environmental conditions.

At present, it is not clear how the AHLs act on nitrifying bacteria in some adverse environmental conditions. One hypothesis is that AHLs can regulate bacterial communities, and thus affect symbiotic relationships and lead to changes in nitrifying bacteria in nitrifying sludge. It was found in this study that the functions and structure of nitrifying bacterial community were highly sensitive to AHLs. When the system was at low temperature or in the presence of DCD, the gene expression produced by the exogenous addition of AHL had been documented either at an individual or community level of bacteria. However, it is worth noting that not all the members of a community were affected similarly by AHLs. In AHLs addition study, the top 36 most abundant community members responded differently to AHLs. This phenomenon had also been observed in other research^21^. In addition, previous studies have shown that AHL-producers were more sensitive to the addition of AHLs. In this study, the abundance of 7 bacteria increased after the exogenous addition of AHLs, including *Nitrosomonas*, *Nitrospira*, *Ferruginibacter*, *Thauera*, *Hyphomicrobium, Mycoplasma* and *Bacillus,* while most of these bacteria did not report the production of signaling molecules. The influence of community structure, function and composition need to be addressed fully in further studies. Several types of AHLs produced by AOB had been demonstrated in supernatant in previous studies^23^. The biofilm formation of *Nitrosomonas europaea* is closely related to QS, and addition of exogenous AHL can help it rapidly recover its activity in a state of starvation. However, no AHL synthase homologues were detected in this bacterium, suggesting that *Nitrosomonas europaea* can utilize AHL and synthesize AHL in another way^12,27^. These researches indicate that the presence of AHLs have the potential to enhance the AHL-incativating activities of AOB under stressful conditions, which were confirmed again in our batch tests on the utilization efficiency of AHLs by bacteria. Although the mechanism behind this phenomenon needs to be further investigated, these results offered a possibility that nitrifying bacteria may increase the usage of AHL to overcome adverse living conditions. But AHL addition method might not work for all conditions. In a good growth state, nitrifying bacteria was not strongly dependent on QS as the NH_4_^+^–N removal efficiency in AHL addition system was no significant increased. Signal molecules are not concentration-dependent, and the appropriate addition of signal molecules may aid the promotion of community stability and pollutant removal^19^. Combined with above results, the AHL addition strategy needs to be considered with other environmental factors, especially pH value.

## Conclusions

This study revealed the potential role of AHLs in nitrifying bacteria community survival under certain adverse conditions (low temperature, low pH level and presence of DCD). The DCD group and low temperature group treated with AHLs had better performance, and the nitrifying bacteria abundance and QS activity were significantly higher than that of the control group. Community analysis and qPCR indicated that QS may potentially play functional roles in community restructuring by strengthening nitrifying bacteria species. However, adding AHLs in normal condition (28 °C, pH = 8) or acid condition (pH 5.5) had no significant change to the system, which may be due to the strong bacterial activity under normal conditions and limited space for further improvement, and the influence of acid conditions on nitrifying bacteria was greater than that of AHLs. Therefore, the regulation strategy of AHLs on nitrification under adverse factors should be considered in combination with the pH value of the system.

## Supplementary Information

Supplementary Information.

## Data Availability

The datasets generated and/or analysed during the current study are not publicly available due to they also form part of an ongoing study but are available from the corresponding author on reasonable request.
