# Supplementary material for: Potential roles of acyl homoserine lactones (AHLs) in nitrifying bacteria survival under certain adverse circumstances
Source: Sci Rep. 2023 Feb 6;13:705. doi: 10.1038/s41598-022-23123-x (PMC9902454; doi:10.1038/s41598-022-23123-x)
Supplement: Supplementary file 1 — Supplementary Information. [file 41598_2022_23123_MOESM1_ESM.doc]

| Compound | Concentration | Trace elements solution | Concentration |
| --- | --- | --- | --- |
| (NH _4_)_2_SO_4_ | x^a^ g/L | Na_2_ EDTA | 4292 mg/L |
| KH_2_PO_4_ | 0.054 g/L | FeCl_2_. 4H_2_O | 1988 mg/L |
| KCl | 0.075 g/L | MnCl_2_. 2H_2_O | 81 mg/L |
| CaCl_2_.2H_2_O | 0.147 g/L | NiCl_2_. 6H_2_O | 24 mg/L |
| MgSO_4_.7H_2_O | 0.049 g/L | CoCl_2_. 6H_2_O | 24 mg/L |
| HEPES buffer | 4x g/L | CuCl_2_. 2H_2_O | 17 mg/L |
| 0.04% Bromothymol blue solution | 5 ml/L | ZnCl_2_ | 68 mg/L |
|  |  | Na_2_MoO_4_. 2H _2_O | 24 mg/L |
| Trace elements solution | 1 ml/L | Na_2_WO_4_. 2H_2_O | 33 mg/L |
|  |  | H_3_BO_3_ | 62 mg/L |

Table S1 The composition of the synthetic feed stock solution

a: Variable amounts depending on strains and experiments.

Table S2 Summary of overall operation data of 4 group systems

| System | N | | A | | D | | L | |
| --- | --- | --- | --- | --- | --- | --- | --- | --- |
|  | BR | ER | BR | ER | BR | ER | BR | ER |
| Temperature | 28°C | 28°C | 28°C | 28°C | 28°C | 28°C | 10°C | 10°C |
| pH | 8.0 | 8.0 | 5.5 | 5.5 | 8.0 | 8.0 | 8.0 | 8.0 |
| AHL addition | 0 | 1 μM | 0 | 1 μM | 0 | 1 μM | 0 | 1 μM |
| DCD addition | 0 | 0 | 0 | 0 | 5 mg/L | 5 mg/L | 0 | 0 |

N: normal group; A: Acid group; D: DCD group; L: low temperature group

BR: Blank reactor; ER: Experiment reactor

Table S3 Primers information used in the qPCR process

| Target | Primer name | Sequence(5’-3’) |
| --- | --- | --- |
| amoA gene | amoA1F | GGGGTTTCTACTGGTGGT |
|  | amoA2R | CCCCTCKGSAAAGCCTTCTTC |
| nxrA gene | nxrA1F | CAGACCGACGTGTGCGAAAG |
|  | nxrA1R | TCYACAAGGAACGGAAGGTC |
| nxrB gene | nxrB-F | TACATGTGGTGGAACA |
|  | nxrB-R | CGGTTCTGGTCRATCA |
| 16s rRNA | BSF8 | AGAGTTTGATCCTGGCTCAG |
|  | BSF534 | ATTACCGCGGCTGCTGG |

Figure S1 The abundance of AOB, NOB and total bacteria in four groups. N: normal group; A: Acid group; D: DCD group; L: low temperature group; BR: Blank reactor; ER: Experiment reactor.
